# Ferroptosis and hyperoxic lung injury: insights into pathophysiology and treatment approaches

**DOI:** 10.3389/fphar.2025.1568246

**Published:** 2025-03-18

**Authors:** Xiaoqiong Zhou, Lei Tian, Wenyan Xiong, Yulan Li, Qian Liu

**Affiliations:** ^1^ Department of Anesthesiology, Zigong First People’s Hospital, Zigong Academy of Medical Sciences, Zigong, China; ^2^ The First School of Clinical Medicine, Lanzhou University, Lanzhou, China; ^3^ Department of Anesthesiology, Yibin Maternity and Children Hospital, Yibin, China; ^4^ Department of Anesthesiology, The First Hospital of Lanzhou University, Lanzhou, China

**Keywords:** hyperoxia, lung injury, ferroptosis, therapy, programmed cell death

## Abstract

Hyperoxia therapy is a critical clinical intervention for both acute and chronic illnesses. However, prolonged exposure to high-concentration oxygen can cause lung injury. The mechanisms of hyperoxic lung injury (HLI) remain incompletely understood, and current treatment options are limited. Improving the safety of hyperoxia therapy has thus become an urgent priority. Ferroptosis, a novel form of regulated cell death characterized by iron accumulation and excessive lipid peroxidation, has been implicated in the pathogenesis of HLI, including diffuse alveolar damage, vascular endothelial injury, and bronchopulmonary dysplasia. In this review, we analyze the latest findings on ferroptosis and therapeutic strategies for HLI. Our aim is to provide new insights for the treatment of HLI and to facilitate the translation of these findings from bench to bedside.

## 1 Introduction

Oxygen (O_2_) therapy is essential for critically or acutely ill patients (Angus, 2020; Helmerhorst et al., 2015). During the COVID-19 pandemic, high-concentration oxygen therapy (hyperoxia, FiO_2_ ≥ 50%) is one of the few available options to treat hypoxemia-related respiratory failure (Mellado-Artigas et al., 2021; Hanidzir and Robson, 2021). However, hyperoxia is a double-edged sword. Overwhelming evidence from preclinical and clinical studies demonstrates that prolonged exposure to high concentrations of O_2_ is associated with unfavorable outcomes, increased morbidity, and mortality (Rothen, 2010; Lilien et al., 2022; Staehr-Rye et al., 2017; Girardis et al., 2016; Hong et al., 2021; Ni et al., 2019). The lungs are particularly vulnerable due to their direct exposure to O_2_. Acute lung injury (ALI) is a major cause of death in patients receiving hyperoxia, and current therapeutic options have limited efficacy. Hyperoxic acute lung injury (HALI) is characterized by alveolar epithelial and pulmonary endothelial damage (Lius and Syafaah, 2022). Hyperoxia is also a well-known cause of chronic lung injuries such as pulmonary interstitial fibrosis and bronchopulmonary dysplasia (BPD)—a chronic lung disease characterized by abnormal lung and pulmonary vascular development (Willis et al., 2020; Tanni et al., 2021; Gilfillan et al., 2021). Despite intensive efforts to elucidate the mechanisms underlying HLI and develop novel therapies, current treatments remain suboptimal due to the poorly understood pathogenesis.

Ferroptosis, a term coined in 2012, is a new form of programmed cell death (PCD) driven by iron-dependent lipid peroxidation on cell membranes, which can be suppressed by iron chelators or small lipophilic antioxidants (Dixon et al., 2012). As a novel form of PCD, ferroptosis is biochemically and morphologically distinct from other classical forms of PCDs, such as apoptosis, necroptosis, pyroptosis, and autophagy (Dixon et al., 2012; Xie et al., 2016). The disruption of iron and lipid metabolism, depletion of antioxidant systems, and mitochondrial dysfunction are the main causes of ferroptosis (Hadian and Stockwell, 2020; Zheng and Conrad, 2020). In recent years, ferroptosis has received increasing attention due to its significant role in various pathological conditions and diseases (Jiang et al., 2021). There is growing evidence that excessive iron accumulation, unbalanced mitochondrial quality control (MQC), reduced antioxidant levels, and increased lipid peroxidation play crucial roles in HLI (Sidramagowda et al., 2020; Mousavi et al., 2011; Ma et al., 2018; Qin et al., 2023). Furthermore, direct evidence suggests that targeting ferroptosis could be an effective therapeutic strategy for treating HLI (Liu C. et al., 2024; Guo et al., 2022; Chou and Chen, 2022a; Jia et al., 2021; Wang et al., 2020).

Given the emerging evidence for the mechanisms of ferroptosis in HLI and the great potential of ferroptosis-targeted therapies for HLI treatment, it is necessary to summarize the latest findings and track the progress in this field. In this paper, we provide a brief overview of the regulatory mechanisms of ferroptosis and summarize the latest research and therapies targeting ferroptosis in HLI. We hope this review can provide an update on advances in HLI pathogenesis and newer therapeutic strategies targeting ferroptosis.

## 2 Mechanisms of ferroptosis and links with HLI

**FIGURE 1:**
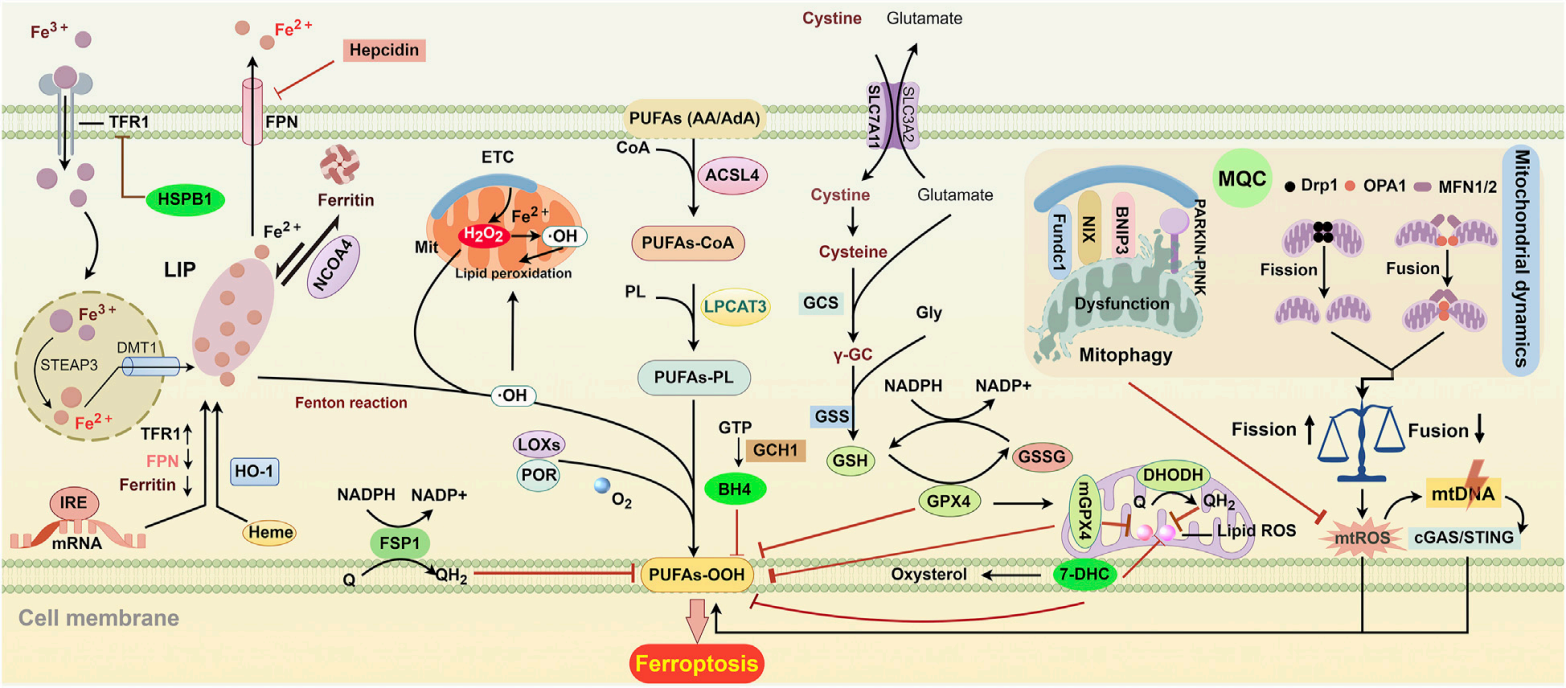
Key regulatory pathways of ferroptosis. ACSL4 and LPCAT3 initiate the biosynthesis of PUFA-PL by incorporating and esterifying PUFAs into phospholipids. Extracellular Fe^3+^ enters cells through TFR-mediated endocytosis. Once inside, it is reduced to Fe^2+^ by the enzyme STAEP3 and then transported to the cytoplasm by DMT1. After the release into the cytoplasm, free iron enters the cytosolic labile iron pool (LIP). Fe^2+^ from the LIP can be stored in ferritin and can be released again through NCOA4-mediated degradation. FPN is responsible for exporting iron to the extracellular space. IRE controls the translation of iron metabolism-related mRNA, including FPN, ferritin, and TFR. The levels of TFR and FPN can also be regulated by HSPB1 and hepcidin, respectively. HO-1 converts heme into biliverdin and iron, which raises intracellular iron levels. Electron leakage from the, ETC., generates O_2_^·−^ and H_2_O_2_, leading to the formation of OH· through Fenton action, which accelerates lipid peroxidation in cellular and mitochondrial membranes. Additionally, LOXs and POR can promote lipid peroxidation by converting PUFAs into hydroperoxides. SLC7A11-mediated cystine transport enhances the synthesis of GSH. GPX4 uses GSH to protect cells from lipid peroxidation and ferroptosis. GSH can enter the mitochondria to form mGPX4, which collaborates with DHODH to help prevent ferroptosis. 7-DHC protects lipids from autoxidation in plasma and mitochondria and prevents ferroptosis. FSP1 is primarily located in plasma membranes, where it converts Q into QH_2_ to eliminate harmful lipid peroxides. GCH1 suppresses ferroptosis by generating the antioxidant BH4. Mitochondrial dynamics and mitophagy are essential for mitochondrial quality control (MQC). Disruption of MQC can lead to ferroptosis by increasing mtROS, which damages mtDNA. This damaged mtDNA can enter the cytosol and trigger lipid peroxidation through the mtDNA/cGAS/STING pathway. Abbreviations: ACSL4, acyl-CoA synthetase long-chain family member 4; BH4, tetrahydrobiopterin; cGAS/STING, cyclic GMP-AMP synthase-stimulator of interferon genes; 7-DHC, 7-Dehydrocholesterol; DHODH, dihydroorotate dehydrogenase; DMT1, divalent metal transporter 1; Drp1, dynamin-related protein 1; FPN, ferroportin; FSP1, ferroptosis suppressor protein 1; Fundc1, FUN14 domain containing 1; GCH1, GTP cyclohydrolase 1; GPX4, glutathione peroxidase 4; GSH, glutathione; GSSG, glutathione disulfide; HO-1, heme oxygenase 1; IRE, iron response element; HSPB1, heat shock protein beta-1; LOXs, lipoxygenase; LPCAT3, lysophosphatidylcholine acyltransferase 3; MFN1/2, mitofusin-1/2; mGPX4, mitochondrial glutathione peroxidase 4; mtDNA, mitochondrial DNA; NADPH, nicotinamide adenine dinucleotide phosphate; NCOA4, nuclear receptor coactivator 4; OPA1, optic atrophy protein 1; PARKIN, Parkin RBR E3 ubiquitin-protein ligase; PINK1, PTEN-induced kinase 1; PL, phospholipid; POR, P450 oxidoreductase; PUFAs, polyunsaturated fatty acids; Q, ubiquinol; STEAP3, six-transmembrane epithelial antigens of the prostate 3; TFR, transferrin receptor.

Ferroptosis was first identified as a novel, iron-dependent form of PCD by Stockwell et al., in 2012. This non-apoptotic process, induced by erastin in tumor cells, exhibits characteristic ultrastructural morphological features including mitochondrial shrinkage, increased membrane density, cristae disappearance, and outer membrane rupture (Dixon et al., 2012). As shown in Figure 1, ferroptosis is triggered mainly by three metabolic disorders: excessive intracellular iron accumulation, increased lipid ROS [particularly hydroxyl radicals (OH·)], and decreased activity of antioxidant systems [especially glutathione peroxidase 4 (GPX4)] (Jiang et al., 2021; Yang and Stockwell, 2016). Iron chelators [such as deferoxamine (DFO) and deferiprone] and some small radical-trapping antioxidants (e.g., α-tocopherol, ferrostatin-1, and liproxstatin-1) can prevent it (Zheng and Conrad, 2020; Stockwell, 2022). Numerous studies have revealed that iron overload, lipid peroxidation accumulation, and antioxidant system imbalance are involved in the pathological mechanisms of hyperoxia-induced acute and chronic lung injury, which are primarily characterized by alveolar epithelial cell injury and BPD, respectively. Therefore, targeting ferroptosis may be a potential strategy for treating HLI.

### 2.1 Iron metabolism and HLI

#### 2.1.1 Iron in ferroptosis

Iron is essential for ferroptosis. The iron-catalyzed Fenton reaction generates reactive OH·, which are primary agents of lipid peroxidation. Additionally, iron-containing enzymes such as lipoxygenases (LOXs) and P450 oxidoreductase (POR) promote lipid peroxidation by catalyzing the oxidation of polyunsaturated fatty acids (PUFAs) to hydroperoxides and by transferring electrons from NADPH to O_2_ (Koppula et al., 2021; Wenzel et al., 2017; Yang et al., 2016; Zou et al., 2020).

Intracellular iron levels are maintained through the balance of uptake, storage, and export processes. Transferrin receptor 1 (TfR1) mediates iron uptake into cells. First, ferric iron (Fe^3+^) binds to transferrin on the cell membrane to form Tf-Fe^3+^, which then enters cells via TfR1-dependent endocytosis. In the endosome, Fe^3+^ is reduced to Fe^2+^ by six-transmembrane epithelial antigen of prostate 3. Finally, Fe^2+^ is released into the cytoplasm by divalent metal transporter 1 (DMT1) for storage or utilization (Chen et al., 2020). Knockdown or degradation of TfR1 significantly reduces intracellular Fe^2+^ levels and prevents ferroptosis (Shao et al., 2024; Zhao et al., 2023). Conversely, elevated expression and stability of TfR1 promote ferroptosis by increasing labile iron accumulation (Yu et al., 2024; Zhou et al., 2024).

Heme oxygenase-1 (HO-1) is another source of intracellular labile iron and can be highly induced by heme and multiple stressors, including oxidative stress, inflammation, and infection (Dunn et al., 2014). HO-1 metabolizes heme into biliverdin, which is rapidly converted to bilirubin, exerting anti-inflammatory and antioxidant effects. Additionally, this process generates Fe^2+^, which increases labile iron levels and promotes lipid peroxidation (Li X. et al., 2024; Huang et al., 2024; Chen C. et al., 2023; Yang et al., 2023). These properties confer a dual role on HO-1 in ferroptosis. Nuclear factor E2-related factor 2 (Nrf2), the most important upstream activator of HO-1, is activated under stressful conditions. The Nrf2/HO-1 axis, a crucial ferroptotic signal transduction pathway, is recognized as the primary defense mechanism against ferroptosis by restoring GPX4 levels and its antioxidant functions (Dodson et al., 2019; Wei et al., 2020). HIF-1α, another critical upstream molecule of HO-1, is activated under hypoxia conditions and primarily mediates pro-ferroptotic effects by causing iron accumulation and lipid peroxidation (Lu et al., 2024; Li et al., 2025; Yang et al., 2025). However, differences in upstream activators do not fully explain the dual role of HO-1 in ferroptosis. Inconsistent findings indicate that the Nrf2/HO-1 axis may promote ferroptosis, while the HIF-1α/HO-1 axis may inhibit it (Wei et al., 2021; Tang et al., 2021; Yao et al., 2024; Shi et al., 2024). Accumulating evidence suggests that HO-1 levels must be tightly regulated for cytoprotective effects. In disorders with long-term HO-1 expression, such as cancer, fibrosis, Alzheimer’s disease, diabetic nephropathy, and cerebral hemorrhage, HO-1 exacerbates ferroptosis by causing Fe^2+^ overproduction and ROS generation (Chang et al., 2018; Ryter and Tyrrell, 2000; Feng et al., 2021; Choi and Kim, 2022; Maus et al., 2023).

Intracellular free iron can join the cytosolic labile iron pool (LIP), a poorly defined dynamic pool of Fe^2+^ bound to low-affinity chelators like glutathione (GSH) and poly (rC)-binding proteins. This unstable binding makes LIP a source of cellular free iron. Iron from the LIP can also be stored in ferritin, a heteropolymer composed of FTH and light ferritin (FTL) chains, providing a relatively safe storage form (Koorts and Viljoen, 2007). Ferritin is considered a protective factor against ferroptosis, whereas its disruption, degradation, or reduced expression increases ferroptosis risk (Zhu et al., 2022; Chittineedi et al., 2023). Ferritin can also be degraded by autophagy, known as ferritinophagy, which is mediated by nuclear receptor coactivator 4 (NCOA4) and increases cellular labile iron content and ferroptosis sensitivity (Jia et al., 2024). On the contrary, NCOA4 knockdown or disruption of the NCOA4-FTH1 interaction inhibits ferritinophagy-mediated ferroptosis by reducing cytosolic iron levels (Wang et al., 2023; Yang et al., 2024; Ji et al., 2024; Gao et al., 2024; Song et al., 2024). Thus, ferritin serves both as an iron source and a protective agent against ferroptosis. Targeting ferritin synthesis and degradation may be an important strategy to modulate intracellular iron levels and control cell fate.

Intracellular iron is primarily transported out of the cell in the divalent form by ferroportin (FPN) (Ward and Kaplan, 2012) or be exported via ferritin-containing multivesicular bodies (MVBs)/exosomes, a process facilitated by prominin 2 (Brown et al., 2019). These mechanisms reduce iron accumulation and enhance cellular resistance to ferroptosis.

TfR1, ferritin, and FPN are key regulators of iron uptake, storage, and export, respectively, and are dominated by iron regulatory proteins (IRPs)-dependent post-transcriptional regulation (Hentze et al., 2010). At a low Fe^2+^ content condition, IRPs bind to the iron response elements (IREs), promoting TfR1 synthesis while inhibiting ferritin and FPN expression. Additionally, TfR1 and FPN levels can be regulated through an IRP-independent manner. Heat shock protein beta-1 (HSPB1) has been shown to inhibit TfR1 expression and reduce intracellular iron concentration, thereby attenuating erastin-induced ferroptosis (Sun et al., 2015). FPN is also regulated by hepcidin, a peptide hormone secreted predominantly by hepatocytes. When hepcidin binds to FPN, it will induce the FPN endocytosis and proteolysis of the FPN-hepcidin complex, thereby modulating iron export (Billesbolle et al., 2020; Nemeth et al., 2004).

Therefore, both direct interventions, such as iron chelation, and indirect strategies targeting iron import, storage, or export, represent promising therapeutic approaches for ferroptosis-related diseases.

#### 2.1.2 A link to HLI

**FIGURE 2:**
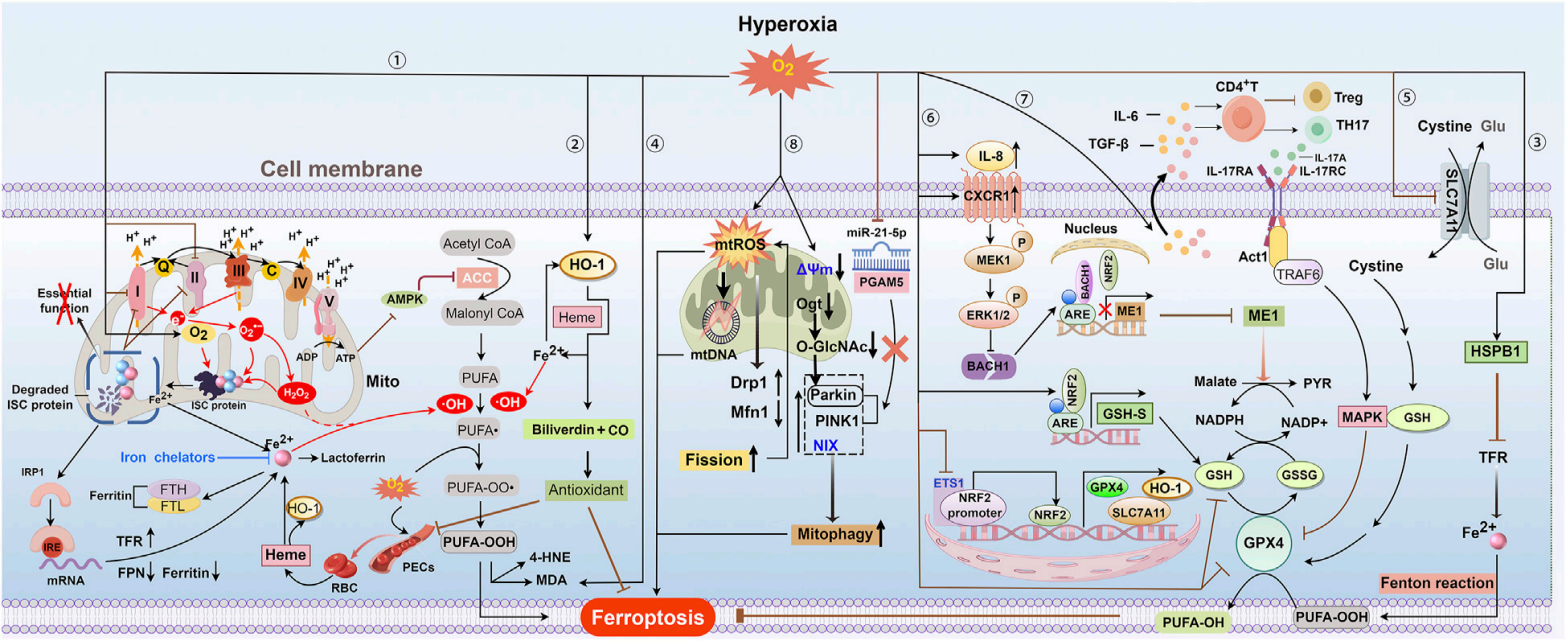
Overview of ferroptosis in hyperoxia. ① and ② Hyperoxia can decrease ATP production by inhibiting oxidative phosphorylation driven by the ETC. This may inhibit ACC by activating AMPK activity through the sensing of the ADP: ATP ratio, which further reduces the synthesis of PUFAs. Hyperoxia disrupts complexes I and II of the ETC, leading to increased production of O_2_^·−^ and H_2_O_2_ due to electron leakage. These reactive species, along with oxygen, can degrade ISC-containing protein complexes, inactivating crucial enzymes and releasing iron. The loss of ISC proteins exacerbates, ETC, dysfunction, further elevating levels of O_2_^·−^ and H_2_O_2_ and causing constant damage to ISC proteins. Reduced ISC levels can boost IRP1 binding to IREs, leading to increased cellular iron uptake by raising TFR expression and lowering ferritin and FPN expression. Excessive H_2_O_2_ promotes the generation of OH· through Fenton reactions and increases the peroxidation of PUFAs in plasma and mitochondrial membranes, ultimately leading to ferroptosis. Exposure to hyperoxia increases the vascular permeability of PECs and leads to the leakage of RBCs and their degradation into heme. The heme-induced HO-1 catabolizes heme to iron HO-1, thereby increasing intracellular iron levels. To reduce HLI, excessive iron can enhance HO-1 gene expression and increase ferritin and lactoferrin levels during hyperoxia, thereby providing an antioxidant effect by producing biliverdin. Excess iron increases HO-1 gene expression, producing biliverdin for antioxidant effects, and raises ferritin and lactoferrin levels during hyperoxia, potentially helping to reduce HLI. HO-1 gene expression can also be induced by hyperoxia itself to exert antioxidant effects. ③ Increased oxygen levels enhance HSPB1 mRNA, which may reduce cellular iron by suppressing TFR expression and thus limit ferroptosis. ④ and ⑤ Hyperoxia exposure will inhibit the SLC7A11-GPX4 axis and increase MDA levels, thereby promoting lipid peroxidation. ⑥ Hyperoxia elevates BACH1 expression, which competes with NRF2 for ME1 promoter binding and transcription. This limits malate oxidation to pyruvate and NADPH-dependent GSH production. Hyperoxia suppresses the ETS1/Nrf2 pathway, resulting in decreased levels of downstream antioxidant components such as HO-1, SLC7A11, and GPX4. Hyperoxia might enhance GSH synthesis via the Nrf2-GSH synthetase pathway as a compensatory mechanism. ⑦Hyperoxia induces HALI by initiating IL-17A-mediated ferroptosis in AEC II through the downregulation of GPX4 via the Act1-TRAF6-p38 MAPK pathway. ⑧ Hyperoxia increases the production of mtROS and causes mtDNA damage, which raises sensitivity to ferroptosis. Additionally, mtROS enhances mitochondrial fission by increasing Drp1 and decreasing Mfn1, causing mitochondrial fragmentation and more mtROS production, which in turn accelerates lipid peroxidation and ferroptosis. Hyperoxic exposure leads to the dissipation of mitochondrial membrane potential (Δψ), loss of ATP, and a burst of ROS. Hyperoxia reduces Ogt and O-GlcNAc levels, enhancing Parkin-dependent mitophagy and HLI. PINK1/Parkin and NIX-mediated mitophagy under hyperoxia contribute to BPD pathogenesis and impaired alveolar development. Hyperoxia reduces miR-21–5p levels, enhancing mitophagy and worsening acute lung injury by directly affecting the PGAM5-related PINK1/Parkin pathway. Abbreviations: AMPK, adenosine monophosphate-activated kinase; ARE, antioxidant response element; C, cytochrome c; CXCR1, C-X-C motif chemokine receptor 1; ETS1, E26 oncogene homolog 1; FTH and FTL, heavy and light ferritin chains; GSH-S, GSH synthetase; 4-HNE, 4-hydroxynonenale; IRE, iron response element; IRP, iron regulatory protein; ISC, iron-sulfur cluster; MAPK, p38 mitogen-activated protein kinase; MDA, malondialdehyde; ME1, malic enzyme 1; Ogt, O-GlcNAc transferase; PECs, pulmonary endothelial cells; PYR, pyruvate; RBC, red blood cell.

It is not surprising that ferroptosis is recognized as a driving factor of HLI because iron-mediated oxidative damage is a hallmark of hyperoxia-induced lung damage. Prior to the introduction of the term ferroptosis, numerous studies had already documented the association between iron- or iron metabolism-related proteins and HLI (Figure 2). For instance, a significant increase in the levels of ferritin light subunit mRNA, which is involved in long-term iron storage, was observed in the lungs following hyperoxic exposure (Ryan et al., 1997). Both ferritin and lactoferrin, which are involved in iron sequestration, were found to increase in lung cells, particularly in alveolar macrophages. This increase contributes to the resistance of hypotransferrinemic mice to HLI (Yang et al., 1999). Additionally, high levels of O_2_ were found to upregulate HSPB1 mRNA levels, which may reduce cellular iron levels by inhibiting TfR1 expression (Deng et al., 2024) (Figure 2). Extracellular iron has also been reported to induce hyperoxia-dependent HO-1 gene expression in pulmonary endothelial cells (PECs), interpreted to be a compensatory mechanism that can be blocked by the iron chelator desferrioxamine (Fogg et al., 1999). These findings highlight the essential roles of host defense mechanisms in controlling iron-mediated oxidative stress and highlight the link between iron metabolism and HLI.

Vascular injuries have been shown to induce iron accumulation due to the degradation of hemoglobin-derived heme (Maus et al., 2023) (Figure 2). This finding aligns with recent evidence that increased vascular permeability and subsequent hemoglobin infiltration are key pathological features of HLI (Baik et al., 2023). Therefore, hyperoxia-induced vascular leakage may exacerbate HLI via iron-mediated oxidative damage or ferroptosis (Figure 2). Earlier studies revealed that iron chelation with DFO significantly improved lung development in newborn rats exposed to hyperoxia, promoting alveolarization and respiratory surface area expansion (Frank, 1991; Blanco and Frank, 1993). More recently, Wang et al. also demonstrated that intravenous DFO significantly mitigates lung injury caused by short-term hyperoxic mechanical ventilation (Wang et al., 2020). Additionally, a controlled before-after study demonstrated that deferasirox, an iron chelator, reduced oxidative injury markers, including lipid peroxidation, DNA damage, and protein oxidation, as well as iron levels in bronchoalveolar lavage fluid under hyperoxic conditions (Mousavi et al., 2011). However, subsequent research by the same group reported inconsistent results (Ahmadi et al., 2013). Given the small number of patients and the uncontrolled nature of this study, a larger, well-designed randomized controlled trial is needed to robustly evaluate the antioxidant effects of deferasirox in HLI.

With the extensive study of O_2_ toxicity, a growing body of literature provides a deeper understanding of how hyperoxia affects iron metabolism and ferroptosis (Figure 2). Hyperoxia disrupts mitochondrial electron transport by inhibiting complexes I and II of the electron transport chain (ETC.), leading to electron leakage that generates superoxide (O_2_^·−^) through univalent reduction of O_2_ and subsequent conversion of O_2_^·−^ to hydrogen peroxide (H_2_O_2_) by superoxide dismutase (SOD). Both O_2_^·−^ and H_2_O_2_ accelerate the degradation of the iron-sulfur cluster (ISC)-containing protein complexes (Dixon and Stockwell, 2014). Recently, Baik et al. showed that normalizing O_2_^·−^ and H_2_O_2_ levels is insufficient to prevent hyperoxia-induced degradation of specific ISC-containing protein complexes, implicating that O_2_ itself is the likely culprit (Baik et al., 2023). Such degradation will lead to the inactivation of essential enzymes (such as respiratory chain activity and gene regulation) and labile iron release (Dixon and Stockwell, 2014). These events further exacerbate, ETC., dysfunction, increasing ROS production and causing cyclic damage to ISC-containing proteins. Additionally, reduced ISC levels can enhance IRP1 binding to IREs, promoting cellular iron uptake by upregulating TfR1 expression and downregulating ferritin and FPN expression (Terzi et al., 2021; Hentze et al., 2010).

Preclinical studies have shown that ferroptosis contributes to the pathogenesis of various types of HLI, including alveolar epithelial injury, vascular endothelial injury, and BPD (Table 1). This process is partially driven by the accumulation of ROS through the iron-driven Fenton reaction, which results from iron deposition or disrupted iron metabolism (Liu L. et al., 2024; Guo et al., 2022; Chou and Chen, 2022a; Jia et al., 2021; Yang et al., 2023; Li X. et al., 2024).

**TABLE 1:** Therapeutic strategies targeting ferroptosis in HLI.

Study/ref.	Intervention/administration scheme	Target	Mechanism	Effect	Experimental model	Oxygen concentration/time
Yin et al. (2023)	Fer-1, via medium, 10 μM for 2h; Nrf2 expression	Lipid ROS, Nrf2	Inhibits lipid peroxidation; upregulates GPX4	Alleviates HALI	Human pulmonary microvascular endothelial cell	95% O_2_ for 24 h and 48 h
Chou et al. (2022a)	Cathelicidin, intraperitoneally, 8 mg/kg on postnatal days from 1 to 6	Iron, GSH and GPX4	Decreases iron deposition; improves GSH levels; and enhances GPX4 activity and expression	Improves hyperoxia-induced HLI	SD rat pups	85% O_2_ from postnatal days 1–7
Liu C. et al. (2024)	MDABP, intraperitoneally, 10 mg/kg	Iron, GSH and GPX4	Decreases the levels of Fe^2+^ and ROS and increases GSH and GPX4 expression	Alleviates hyperoxia-induced AEC injury	Newborn SD rats	85% O_2_ from postnatal days 1–7
Yang et al. (2023)	ETS1 overexpression	Nrf2 signaling	Increases the expression of HO-1, SLC7A11, and GPX4; raises GSH levels and decreases Fe^2+^ levels	Ameliorates hyperoxia-induced BPD	Newborn mice	85% O_2_ from postnatal days 1–14
Guo et al. (2022)	Salidroside, once orally every day, 100 mg/kg	GPX4, iron	Increases GPX4 expression and decreases the Fe^2+^ level	Attenuates HALI	Eight-week-old KM mice	90% O_2_ for 24 h
Deng et al. (2024)	Quercetin, via medium, 20 μM for 2 h	PTGS2	Decreases PTGS2 protein expression	Alleviates hyperoxia-induced BPD	HUVECs and BEAS-2B cells	85% O_2_ for 12 h
Liu et al. (2023)	Wedelolactone, intraperitoneally, 50 mg/kg for 6 h	GSH and GPX4	Increases GSH and GPX4 expression	Attenuates HALI	C57BL/6J mice	>90% for 48 h
Li K. et al. (2024)	Wedelolactone, intraperitoneally, 20 mg/kg per day for 3 days	Nrf2 signaling	Upregulates HO-1, SLC7A11, and GPX4 expression; increases GSH levels; reduces Fe^2+^ accumulation by increasing FTH1 and decreasing TfR1 expression levels	Alleviates HALI	C57BL/6J mice; MLE-12 cells	Mice, 99% O_2_ for 72 h; MLE-12 cells, 95% O_2_ for 12, 24, and 48 h

Abbreviations: AEC, alveolar epithelial cell; BEAS-2B, bronchial epithelial cells; BPD, bronchopulmonary dysplasia; Fer-1, ferrostatin-1; GPX4, glutathione peroxidase 4; GSH, glutathione; HALI, hyperoxic acute lung injury; HLI, lung injury; HO-1, heme oxygenase-1; HUVECs, human umbilical vein endothelial cells; MDABP, milk-derived anti-BPD, peptide; PTGS2, prostaglandin-endoperoxide synthase 2; ROS, reactive oxygen species; SD, sprague dawley; xCT, System XC-.

In summary, these findings provide a foundation for clinical investigations into the use of iron chelators in HLI treatment and highlight the need for future research on iron-centered mechanisms of ferroptosis in HLI.

### 2.2 GPX4-GSH anti-ferroptosis pathway and HLI

#### 2.2.1 GPX4-GSH in ferroptosis

Among the anti-ferroptotic defense systems, the GPX4-GSH system plays a predominant role (Figure 1). Cystine, an essential amino acid, is required for the biosynthesis of the antioxidant GSH (Lu, 2009). After being transported into the cell by the solute carrier family 7 member 11 (SLC7A11), cystine is used for GSH biosynthesis via cystine reductase, gamma-glutamyl-cysteine synthetase, and glutathione synthetase (GSH-S) (Figure 1). GPX4 utilizes GSH to convert hydroperoxide to non-toxic lipid alcohols, thereby preventing ferroptosis. This process involves the transition of GSH to oxidized glutathione (GSSG), which can be recycled back to GSH by nicotinamide adenine dinucleotide phosphate (NADPH)-dependent GSH reductase (Figure 1). Inhibition of GSH synthesis through SLC7A11 blockade or deletion (Dixon et al., 2012; Badgley et al., 2020) or inactivation of GPX4 via the GPX4 inhibitor RSL3 can induce ferroptosis (Jiang et al., 2021). Therefore, the GPX4-GSH axis is a potential therapeutic target in ferroptosis-associated diseases.

#### 2.2.2 A link to HLI

Recent studies indicated that hyperoxia inhibits the expression and activity of GSH and GPX4 through multiple mechanisms (Figure 2). Evidence from transcriptome sequencing indicates that HALI is closely associated with ferroptosis and GSH metabolism pathways (Qin et al., 2024). In a BPD animal model, hyperoxia significantly reduced GSH and GPX4 levels while increasing malondialdehyde (MDA) levels, a primary product of lipid peroxidation (Ozdemir et al., 2016; Kennedy and Lane, 1994). These changes collectively contribute to the development of HLI. Beyond inducing chronic lung injury, hyperoxia also promotes inflammation and exacerbates ALI by reducing glutathione peroxidase and SOD activities, while simultaneously increasing lipid hydroperoxide levels (Tao et al., 2012; Nagato et al., 2009).

Nrf2 is a crucial transcription factor in the body’s antioxidant defense. Under oxidative stress or inflammation conditions, Nrf2 can bind to antioxidant response elements and activate the expression of various antioxidant genes, such as HO-1, SLC7A11, and GPX4 (Niture et al., 2014) (Figure 2). Nrf2 has been shown to prevent lipid peroxidation and ferroptosis in various diseases like pulmonary fibrosis and LPS-induced lung injury by regulating GSH synthesis and metabolism (Ai et al., 2024; Dodson et al., 2019; Wang C. L. et al., 2024). It has been demonstrated that hyperoxia increases lung GSH synthesis expression via an Nrf2-GSH-S-dependent pathway, which can relieve HALI to some extent (Reddy et al., 2009; Audi et al., 2022) (Figure 2). However, it remains uncertain whether this response is adaptive or compensatory. In addition to regulating GSH synthesis, Nrf2 is involved in NADPH-dependent reduction of GSSG to GSH. Qin et al. demonstrated that hyperoxia inhibits BACH1 degradation that hyperoxia inhibits BACH1 degradation via the MEK1/ERK axis, which is activated by CXCR1/IL-8 signaling. Elevated BACH1 then competes with Nrf2 for binding to the malic enzyme 1 (ME1) promoter, thereby reducing ME1 transcription. This reduction will reduce NADPH production by limiting malate oxidation to pyruvate, thereby exacerbating HALI by lowering GSH levels and causing redox imbalance in PECs (Qin et al., 2023) (Figure 2).

In addition to the indirect evidence, there is growing direct evidence that the dysfunctional GPX4-GSH pathway contributes to HLI by promoting ferroptosis. Four studies have indicated that hyperoxia decreases GSH and/or GPX4 levels, as well as GPX4 activity, without clarifying the underlying mechanisms (Liu R. et al., 2024; Chou and Chen, 2022a; Chou and Chen, 2022b; Liu et al., 2023). Another study linked GSH reduction to hyperoxia-induced SLC7A11 inhibition but did not elucidate the observed decrease in GPX4 levels (Jia et al., 2021). Subsequently, Yin et al. identified Nrf2 as an upstream regulator of GPX4, which prevents ferroptosis and alleviates HLI (Yin et al., 2023). Additionally, Li et al. found that hyperoxia inhibits Nrf2 expression, leading to the downregulation of SLC7A11 and GPX4 and the depletion of GSH (Li K. et al., 2024). E26 oncogene homolog 1 (ETS1) can bind to the Nrf2 promoter region, promoting its transcription. Under hyperoxia, the ETS1/Nrf2 pathway is inhibited, leading to reduced levels of its downstream components HO-1, SLC7A11, and GPX4. This suppression subsequently induces ferroptosis in type II alveolar epithelial (AEC II) cells (Yang et al., 2023) (Figure 2). These findings suggest that Nrf2 is a potential target for treating HLI. In addition to Nrf2, the p38 mitogen-activated protein kinase (p38 MAPK) is involved in ferroptosis in HALI by mediating GPX4 inactivation. p38 MAPK is a key member of the MAPK family and plays a crucial role in regulating inflammatory mediators and ferroptosis (Yong et al., 2009; Hattori et al., 2017). Guo et al. found that hyperoxia exposure increases IL-6 and TGF-β1 levels in AEC II cells, triggering IL-17A release from immune cells. This, in turn, activates the Act1-TRAF6-MAPK pathway, downregulating GPX4 and inducing ferroptosis in HALI (Guo et al., 2022) (Figure 2).

Collectively, these results indicate that impairment of the GPX4-GSH pathway is a critical driver of ferroptosis in various HLIs and a potential common therapeutic target.

### 2.3 PUFAs in ferroptosis and its link to HLI

PUFAs, such as arachidonic acid and adrenic acid, play a critical role in lipid peroxidation during ferroptosis due to their weak C-H bonds between adjacent C-C double bonds (Conrad and Pratt, 2019). To induce ferroptosis, PUFAs must be incorporated into membrane phospholipids (PLs) to form PUFA-PLs. This process is facilitated by acyl-CoA synthetase long-chain family member 4 (ACSL4), which attaches CoA to PUFAs, and lysophosphatidylcholine acyltransferase 3 (LPCAT3), which integrates PUFA-CoA intermediates into PLs (Doll et al., 2017; Kagan et al., 2017) (Figure 1). ACSL4 deficiency has been reported to inhibit the generation of lipid peroxides and the ferroptosis process (Cui et al., 2021).

Acetyl-CoA carboxylase (ACC) catalyzes the conversion of acetyl-CoA to malonyl-CoA, a key step in the biosynthesis of long-chain fatty acids and the elongation of essential fatty acids, including linoleic and linolenic acids. This process facilitates the formation of longer-chain PUFAs (Beckers et al., 2007). In addition, ACC is also involved in the synthesis of monounsaturated fatty acid (MUFA), which is considered a protective factor in ferroptosis. ACSL3 converts MUFA to MUFA-CoA, which displaces PUFA from phospholipids and inhibits lipid peroxidation (Papsdorf et al., 2023). ACC is regulated by AMP-activated kinase (AMPK), which senses the ADP:ATP ratio and is activated by energy depletion, such as glucose deprivation or hypoxia. Activated AMPK inhibits ACC through phosphorylation, thereby suppressing PUFA synthesis and ferroptosis (Lee et al., 2020). Whether AMPK activation alone can prevent ferroptosis remains to be determined, given its opposing effects on PUFA and MUFA synthesis.

Mitochondria are the primary organelles for ATP production. Hyperoxia inhibits complexes I and II, dissipates mitochondrial membrane potential, and reduces mitochondrial oxidative phosphorylation and ATP production in alveolar epithelial cells, PECs, and lung fibroblasts (Ratner et al., 2009; Das, 2013; Dejmek et al., 2018; Hossain and Eckmann, 2023). These effects may trigger mitochondrial ROS release and activate AMPK, thereby contributing to HLI through the regulation of ferroptosis-associated lipid metabolism and ROS generation (Figure 2).

### 2.4 MQC and HLI

#### 2.4.1 MQC in ferroptosis

Mitochondrial dysfunction is associated with increased production of mitochondrial ROS (mtROS) and mitochondrial lipid peroxidation, both of which are key events in ferroptosis (Tian et al., 2024). MQC is essential for maintaining mitochondrial functionality and integrity, thereby promoting cellular survival. Mitochondrial dynamics and mitophagy are crucial components of MQC. Mitochondrial dynamics involve fusion and fission processes regulated by four key proteins: mitofusin-1 (MFN1), mitofusin-2 (MFN2), and optic atrophy protein 1 (OPA1) promote fusion, while dynamin-related protein 1 (Drp1) drives fission (Chen W. et al., 2023) (Figure 1). Mitochondrial fusion facilitates the mixing of partially compromised mitochondria, preserving their normal morphology and function. Conversely, mitochondrial fission favors the generation of smaller mitochondria, enhancing the removal of damaged mitochondria via mitophagy (Adebayo et al., 2021). However, excessive fission and reduced fusion can cause mitochondrial fragmentation and increased mtROS production, which elevate ROS levels and induce lipid peroxidation and ferroptosis (Chen Z. et al., 2023; She et al., 2021; Shi et al., 2022; Chu et al., 2023). Additionally, mtROS can damage mitochondrial DNA (mtDNA), triggering inflammation or autophagy via the cGAS/STING pathway and thereby inducing ferroptosis (Chen W. et al., 2023; Li et al., 2021a) (Figure 1). However, the relationship between ferroptosis and mitochondrial fission/fusion is complex, as these processes can either promote or inhibit ferroptosis by influencing ROS production and lipid peroxidation (Li et al., 2023; Li et al., 2021b).

Mitophagy is a cellular process that selectively degrades mitochondria through the autophagic machinery. It commonly acts as a protective mechanism by eliminating aged, dysfunctional, or damaged mitochondria to limit ROS production (Youle and Narendra, 2011). The PTEN-induced putative kinase 1 (PINK1)/Parkin pathway is crucial for mitophagy. PINK1, a mitochondrial kinase, targets damaged mitochondria to activate Parkin, an E3 ubiquitin ligase, which then ubiquitinates mitochondrial outer membrane proteins to initiate autophagy. Other receptors, such as NIX, BNIP3, and FUNDC1, also contribute to mitophagy (Rodger et al., 2018) (Figure 1). Mitophagy has been shown to inhibit ferroptosis in various pathological conditions, including cisplatin-induced kidney injury, septic cardiac dysfunction, neurodegenerative disorders, and ischemic injuries (Lin et al., 2023; Liu C. et al., 2024; Wang X. X. et al., 2024; Chu et al., 2023). The underlying mechanisms involve reducing mtROS and lipid peroxidation, mitigating GPX4 downregulation by decreasing excessive ROS release and HO-1 expression, inhibiting the mitochondria-localized AMPK-Parkin-ACSL4 signaling pathway, and activating P62-KEAP1-Nrf2 axis to maintain iron and redox homeostasis. However, excessive mitophagy has been found to promote ferroptosis by releasing labile iron, peroxidized lipids, and ROS (Li et al., 2023; Basit et al., 2017; Yu et al., 2022; Wang et al., 2022). Therefore, both inefficient and excessive mitophagy can be harmful. The findings suggest that modulating mitophagy could be a potential therapeutic strategy for ferroptosis-associated diseases.

#### 2.4.2 A link to HLI

Mitochondrial dysfunction is a key characteristic of HLI (Figure 2). Ma et al. demonstrated that hyperoxia leads to mtROS-induced mitochondrial fragmentation in PECs by upregulating the pro-fission protein Drp1 and downregulating the pro-fusion protein MFN1 (Ma et al., 2018). Conversely, hyperoxia also increases OPA1 expression, which may act as a compensatory mechanism to limit mitochondrial fragmentation. Moreover, hyperoxia increases mtDNA damage and the expression of pro-autophagy proteins while decreasing PINK1 levels in PECs, suggesting impaired autophagy (Ma et al., 2018). This aligns with subsequent findings that hyperoxia triggers autophagy but inhibits autophagosome clearance, leading to impaired mitochondrial function and barrier integrity in lungs and cultured PECs (Beyer et al., 2021). However, in this research, mitophagy remained unchanged, as hyperoxia raised PINK1 expression but not Parkin. Similarly, another study reported that endothelial PINK1 mediates protective effects during hyperoxia, but Parkin expression was not examined (Zhang et al., 2014). Liu et al. showed that pharmacological activation of mitophagy can reduce hyperoxia-induced AEC II injury in BPD models, whereas hyperoxia alone did not affect mitophagy (Liu R. et al., 2024). Conversely, accumulating evidence suggests mitophagy may aggravate hyperoxia-induced acute and chronic AEC II injury (Yu et al., 2020; Liu et al., 2020; Yu et al., 2023) (Figure 2). Therefore, further studies are needed to evaluate mitophagy flux and its relationship with hyperoxia. Given the close association between MQC and ferroptosis, targeting MQC holds great promise for treating HLI.

## 3 Therapeutic strategies targeting ferroptosis in HLI

### 3.1 Current therapy for ferroptosis-associated HLI

#### 3.1.1 Pharmacological inhibitor of lipid peroxidation

As described above, ferroptosis-related mechanisms, such as dysregulation of iron and PUFA metabolism, imbalance in the GPX4-GSH anti-ferroptotic defense system, and MQC, play an important role in HLI. Several specific ferroptosis inhibitors with distinct mechanisms have been identified (Gu et al., 2023). These include iron chelators (DFO, deferiprone), radical-trapping antioxidants (ferrostatin-1, liproxstatin-1), phenothiazine derivatives, ACSL4 inhibitors, and lipoxygenase inhibitors. However, research on ferroptosis inhibitors for HLI treatment is limited, with only one animal study reporting that ferrostatin-1 alleviates HALI by reducing membrane lipid peroxidation (Yin et al., 2023) (Table 1). Thus, additional evidence is needed to further explore this area. Moreover, the development of ferroptosis inhibitors remains in its early stages, with no small molecules yet employed clinically (Gu et al., 2023). This may be attributed to their low efficacy, inadequate selectivity, and severe toxicity.

#### 3.1.2 Bioactive peptides

Cathelicidin, an antimicrobial peptide in the innate immune system, exhibits anti-inflammatory and antioxidant activities (Koon et al., 2011; Ta et al., 2017). Additionally, cathelicidin synthesized in the lung epithelium has antifibrotic effects (Golec et al., 2022; Bals et al., 1998). Recently, bioactive peptides derived from human milk have demonstrated antimicrobial, immunoregulatory, and antioxidant properties (Wada and Lonnerdal, 2020). Ferroptosis, as previously reported, is closely related to redox imbalance and inflammation (Chen C. et al., 2023; Yu et al., 2021; Chen et al., 2024; Stockwell, 2022). These studies demonstrate that cathelicidin and human milk-derived peptides could potentially be used as a therapy for ferroptosis-associated injuries. Indeed, two studies have shown that cathelicidin and a newly identified human milk-derived peptide MDABP can improve lung development and reduce inflammation in neonatal rats (Chou and Chen, 2022b; Liu L. et al., 2024). This is achieved by inhibiting ferroptosis via decreased iron deposition and ROS levels, enhanced GSH levels, and increased activity and expression of GPX4 (Table 1).

Although cathelicidin shows therapeutic potential in preclinical studies for HLI treatment, its clinical application is limited by high synthesis costs, potential toxicity, and poor *in vivo* stability and targetability (Dzurova et al., 2024). Developing effective drug delivery systems to enhance stability and targeting is critical for clinical application.

#### 3.1.3 Gene therapy

ETS1 is a key transcription factor involved in cell proliferation and survival (Kagan et al., 2017). Yang et al. found that ETS1 overexpression alleviates hyperoxia-induced BPD by reducing ROS, MDA, and Fe^2+^ levels through enhancing Nfr2 transcription and activating downstream anti-ferroptotic pathways such as HO-1, SLC7A11, and GPX4 (Yang et al., 2023) (Figure 2). In addition, Yin et al. demonstrated that suppressing GPX4 by Nrf2 inactivation triggers ferroptosis and promotes HLI in human pulmonary microvascular endothelial cells (Yin et al., 2023). Therefore, genetic activation of Nfr2 expression may alleviate HLI by inhibiting ferroptosis.

Despite its potential, gene therapy targeting ETS1 and Nrf2 faces several limitations. A primary concern is the potential for off-target effects, as these genes are part of complex networks beyond ferroptosis, and their overexpression might disrupt other pathways. While short-term studies show benefits, the long-term efficacy and safety of gene therapy for HLI remain to be established. Addressing these limitations will be crucial for advancing gene therapy as a viable treatment option for HLI.

#### 3.1.4 Natural bioactive products

Most research on treating ferroptosis-associated HLI focuses on natural bioactive products, such as salidroside, quercetin, and wedelolactone (Table 1). Salidroside, the bioactive constituent of Rhodiola rosea, exhibits protective effects on multiple organs, including the lungs, through immunomodulatory and antioxidant mechanisms (Liu et al., 2014; Tang et al., 2016; Cao et al., 2022; Zhang et al., 2009). Guo et al. showed that salidroside prevents IL-17A-mediated ferroptosis in lung epithelial cells by regulating the Act1-TRAF6-p38 MAPK-GPX4 pathway, thereby alleviating HALI (Guo et al., 2022) (Figure 2).

Quercetin, a natural flavonoid found in many fruits and vegetables, exhibits potent anti-inflammatory and antioxidant effects (Qi et al., 2022; Xu et al., 2019). It has been shown to alleviate ferroptosis in various diseases by activating antioxidant defenses, including SLC7A11/GSH/GPX4, HO-1, Nrf2, and SOD2 (Wang et al., 2021; Li et al., 2020; Lin et al., 2022). Oxidative stress and inflammation are key mechanisms in BPD. Deng et al. demonstrated that quercetin alleviates hyperoxia-induced BPD by inhibiting ferroptosis through the MAPK/prostaglandin-endoperoxide synthase 2 (PTGS2) signaling pathway (Deng et al., 2024). Although PTGS2 is a marker of ferroptosis, its role remains controversial. Yang et al. showed that upregulated PTGS2 only signifies the onset of ferroptosis without affecting its progression (Yang et al., 2014). Conversely, Xiao et al. demonstrated that miR-212–5p reduces ferroptotic neuronal death following traumatic brain injury by targeting PTGS2 (Xiao et al., 2019). Another study also found that ferroptosis is linked to PTGS2-encoded cyclooxygenase and subsequent inflammatory processes (Araujo et al., 2018). Whether PTGS2 is a target or merely a downstream marker of ferroptosis remains to be further researched.

Wedelolactone, the primary component of Eclipta prostrata, exhibits various pharmacological effects, including anti-inflammatory, antioxidant, and free radical-scavenging properties (Tu et al., 2021). Fan et al. demonstrated that wedelolactone ameliorates acute pancreatitis-associated lung injury through GPX4-mediated mechanisms, thereby reducing lung failure and ferroptosis (Fan et al., 2021). Liu et al. showed that wedelolactone mitigates HALI by inhibiting ferroptosis through increasing GSH and GPX4 levels (Liu et al., 2023). Subsequently, the same team identified Nrf2 as the upstream regulator of the GPX4-GSH defense system (Li K. et al., 2024). Wedelolactone was also found to activate the Nrf2/HO-1 antioxidant pathway and lower Fe^2+^ levels by increasing FTH1 and decreasing TfR1 expression (Li X. et al., 2024). These mechanisms work together to inhibit ferroptosis and alleviate HALI.

To date, none of these natural products (quercetin, salidroside, or wedelolactone) have been evaluated in clinical trials specifically for HLI treatment. Among them, quercetin stands out as the most extensively studied in human trials, demonstrating therapeutic benefits in idiopathic pulmonary fibrosis, chronic obstructive pulmonary disease, and COVID-19 (Mirza et al., 2023). However, its clinical translation faces a critical challenge: poor water solubility leading to low oral bioavailability—a limitation shared by salidroside and wedelolactone (Liang et al., 2021; Guan et al., 2021). These compounds’ polar molecular structures and lack of tissue targeting significantly reduce cellular permeability, often necessitating high doses that may elevate toxicity risks. Structural modifications to enhance metabolic stability and bioavailability are therefore imperative for their pharmaceutical development.

Preclinical and clinical data indicate that quercetin and salidroside are generally well-tolerated, with minimal adverse effects in most populations. However, caution is warranted when using these compounds in polypharmacy scenarios due to potential drug interactions in pharmacokinetics and pharmacodynamics (Liang et al., 2021; Günal-K Ro Lu et al., 2025). The safety profile of wedelolactone remains largely uncharacterized, with critical gaps in understanding its effects on human genetics, protein networks, and metabolism.

In summary, while these natural products demonstrate efficacy in experimental HLI settings, their physiological processing in humans may differ substantially from animal models due to species-specific metabolic pathways. Furthermore, the limited natural abundance of these compounds complicates large-scale isolation for rigorous clinical testing. To bridge these gaps, future efforts should prioritize multicenter randomized controlled trials to validate their efficacy and safety in diverse patient populations, alongside chemical engineering strategies to optimize drug-like properties.

### 3.2 Potential anti-ferroptotic therapy in HLI

In addition to the strategies listed in Table 1, other drugs or interventions for HLI treatment may also exert their effects through anti-ferroptotic mechanisms, as their protective actions partially overlap with those of ferroptosis (Ozdemir et al., 2016; Tao et al., 2012; Liu L. et al., 2024; Yu et al., 2023; Liu et al., 2020).

#### 3.2.1 Targeting GPX4-GSH antioxidant defense

Adrenomedullin, a potent vasodilatory peptide discovered in 1993, has been increasingly recognized for its multiple roles in modulating the inflammatory response (Elsasser and Kahl, 2002). Tao et al. demonstrated that continuous intravenous infusion of adrenomedullin (0.1 μg/kg/min) attenuates HALI by suppressing oxidative stress and inflammation via enhancement of both glutathione peroxidase (GPX) and SOD activities (Tao et al., 2012).

Dexpanthenol is an alcoholic analog of pantothenic acid (vitamin B5) with antioxidant and anti-inflammatory properties. Ozdemir et al. showed that dexpanthenol (500 mg/kg, intraperitoneally) mitigates lung damage in a BPD animal model, partly by increasing GSH and GPX levels (Ozdemir et al., 2016).

GSH and GPX4 are primary defenses against lipid peroxidation and ferroptosis. Adrenomedullin and dexpanthenol may serve as promising anti-ferroptotic treatments for HLI. However, due to a paucity of direct evidence supporting these assumptions, further research is needed to explore the direct interactions between adrenomedullin, dexpanthenol, and ferroptosis in HLI from both clinical and preclinical perspectives.

#### 3.2.2 Targeting mitophagy

Itaconic acid, an unsaturated dicarboxylic acid commonly synthesized from cis-aconitate in the TCA cycle (O'Neill and Artyomov, 2019), exhibits anti-inflammatory properties in the context of severe infections and tissue damage (Lampropoulou et al., 2016). Recently, Liu et al. found that itaconic acid alleviates hyperoxia-induced BPD by promoting TFEB-mediated mitophagy, which helps remove dysfunctional mitochondria and reduces apoptosis in AEC II cells (Liu R. et al., 2024).

O-linked N-acetylglucosamine glycosylation (O-GlcNAcylation) is a posttranslational modification catalyzed by O-GlcNAc transferase (OGT). Yu et al. found that increased O-GlcNAcylation disrupts mitochondrial homeostasis by promoting Parkin-dependent mitophagy in hyperoxia-induced ACE II cell injury (Yu et al., 2023). This effect can be mitigated by the OGT inhibitor OSMI-1 (Figure 2).

PGAM5, a 32 kD mitochondrial protein from the phosphoglycerate mutase family, has been identified as a new mitophagy regulator in the PINK1/Parkin pathway (Lu et al., 2014). Liu et al. showed that miR-21–5p alleviates HALI by inhibiting PINK1/Parkin-dependent mitophagy and mitochondrial damage via direct binding and silencing PGAM5 (Liu et al., 2020) (Figure 2).

Given the dual role of mitophagy in ferroptosis, pharmacological or genetic manipulation of mitophagy may serve as a potential therapeutic strategy for HLI. However, in clinical practice, determining whether mitophagy promotes or inhibits ferroptosis remains challenging. Mitochondrial transplantation may provide a potential solution to this problem.

## 4 Limitations and perspectives

First, most studies have focused on ferroptosis in neonatal models of hyperoxia-induced BPD. This limitation may affect the generalizability to other populations and types of HLI. Second, ferroptosis mechanisms remain incompletely understood, with only a few identified as contributing to HLI. Therefore, further research is required to enhance the evidence base in this area. Third, the severity and mortality of HLI vary among animal species and do not accurately predict human responses to oxygen toxicity. Fourth, although natural bioactive products show promise due to multiple anti-ferroptotic targets and relatively high safety, they are currently limited to preclinical HLI studies due to their low bioavailability, potential toxicity, and complex pharmacological actions. Future efforts should prioritize optimizing drug-like properties and conducting multicenter trials to validate efficacy and safety. Furthermore, the heterogeneity of HLI may influence the translation of findings from animal models to clinical practice. Acute HLI is primarily characterized by inflammation and loss of the antioxidant defense system whereas chronic HLI involves iron deposition in addition to the aforementioned mechanisms. Cellular heterogeneity has also been observed. Hyperoxia-induced injury in PECs is closely associated with mitochondria-derived ferroptosis, which includes reduced production of NADPH and GSH, impaired mitochondrial oxidative phosphorylation and ATP production, and disrupted mitochondrial dynamics. Future research should focus on elucidating these distinct mechanisms to develop targeted therapies for different types of HLI.

## 5 Conclusion

In this review, we summarize the direct and indirect evidence of ferroptosis in HLI pathology. Under hyperoxia, mitochondria serve as both a primary source of ROS and a driver of iron accumulation. Hyperoxia inhibits the, ETC., reducing ATP production and increasing mtROS generation. Additionally, mtROS disturb MQC and exacerbate mtDNA damage, further increasing ROS production. They also contribute to iron overload by degrading ISC proteins, upregulating TFR expression, and downregulating ferritin and FPN through IRP-dependent post-transcriptional regulation. Hyperoxia-induced vascular leakage results in iron accumulation via heme degradation from hemoglobin. In addition to promoting excessive ROS and iron accumulation, hyperoxia also suppresses antioxidant defenses by inhibiting Nrf2 expression, reducing Nrf2 binding to the ME1 promoter, mediating GPX4 deletion via p38 MAPK, and inhibiting the cystine/glutamate antiporter. Collectively, these mechanisms contribute to lipid peroxidation and ferroptosis in HLI.

Hyperoxia also activates compensatory mechanisms to mitigate lung injury by reducing iron overload and alleviating oxidative stress. These mechanisms involve upregulating HSPB1 mRNA, suppressing TfR expression, increasing lactoferrin levels, and enhancing HO-1 expression and GSH synthesis.

The Nrf2 signaling pathway may play an important role in HLI therapy due to its ability to activate multiple antioxidant genes and regulate Fe^2+^ levels. Among the ferroptotic mechanisms, MQC, iron metabolism, and GPX4-GSH defense system occupy a central position in HLI pathogenesis. Future therapeutic strategies should focus on restoring mitochondrial integrity, modulating iron homeostasis, and enhancing antioxidant capacity to mitigate hyperoxia-induced ferroptosis. Multi-target anti-ferroptotic therapies, especially natural bioactive products, hold great promise for HLI treatment. However, translating these preclinical findings into clinical practice remains challenging and requires further research.
